# Optimizing the application of potassium sulfate fertilizer based on the variation in soil mineral accumulation and tobacco growth: a 10-year field experiment

**DOI:** 10.3389/fpls.2025.1748377

**Published:** 2026-01-12

**Authors:** Keke Yu, Zhenwang Zhang, Tao Liu, Huijie Fu, Penghui Lv, Zhe Zhang, Ping Cong, Huaixu Zhan, Lina Tang, Zhiyuan Li, Jianxin Dong

**Affiliations:** 1 Key Laboratory of Tobacco Biology and Processing, Ministry of Agriculture and Rural Affairs, Tobacco Research Institute of Chinese Academy of Agricultural Sciences, Qingdao, China; 2 Engineering Research Center of Plant Growth Regulator, Ministry of Education, College of Agronomy and Biotechnology, China Agricultural University, Beijing, China; 3 China Tobacco Jiangsu Industrial Co., Ltd., Nanjing, China; 4 China Tobacco Hebei Industrial Co., Ltd., Shijiazhuang, China; 5 Tobacco Science Research Institute, Fujian Tobacco Monopoly Administration, Fuzhou, China

**Keywords:** biomass, correlation, K_2_SO_4_ fertilizer, potassium content, soil pH

## Abstract

Potassium (K) is an essential element in tobacco production, especially in Shandong, the main tobacco-growing province in China, where the improvement of tobacco yield and quality is limited by soil K deficiency, thus potassium sulfate (K_2_SO_4_) fertilizer is popularly applied by farmers. However, it is unknown how soil physicochemical characteristics and tobacco growth respond to the utilization of K_2_SO_4_ fertilizer in the long term. This study aimed to determine the optimal application amount of K_2_SO_4_ fertilizer to improve soil health and enhance tobacco leaf K content without yield loss. Four treatments were tested, namely mixes of 82.5 kg a.i. ha ^−1^ K_2_O + 66.0 kg a.i. ha ^−1^ sulfur (K_82.5_ + S_66_), 165.0 + 95.7 (K_165_ + S_95.7_), 247.5 + 125.4 (K_247.5_ + S_125.4_) and no fertilizer (CK). Excessive use of K_2_SO_4_ fertilizer led to soil acidification and a significant decrease in arylsulfatase activity. Soil available potassium (AK) and pH, that exhibited diverse responses over course of 10 years, increased and decreased with the amount of K_2_SO_4_ fertilizer applied, respectively. Moreover, the K content in tobacco plants significantly increased after K_2_SO_4_ fertilizer application, with that in tobacco leaves being 162.3% and 45.2% higher than that in tobacco roots and stems, respectively. Soil AK exhibited a significant positive correlation with the K content in tobacco roots, stems, and leaves, while soil pH showed the opposite performance. Soil pH is a pivotal factor influencing soil physicochemical characteristics, total dry matter and the K content in tobacco tissue. The application of K_165_ + S_95.7_ was more reasonable and economical in terms of matter accumulation. We concluded that the optimal application rate in Shandong was K_165_ + S_95.7_, which not only improved soil health and tobacco leaf K content but also reduced input without causing yield loss.

## 1 Introduction

Tobacco (*Nicotiana tabacum* L.), as a crucial economic crop, has generous benefits for farmers and the Chinese government, as its planting area and tobacco production play a vital role in the world (Ti et al., 2024). In 2022, the tobacco yield per unit area in China reached 2078.8 kg ha^−1^, and the flue-cured tobacco planting area measured 1.0 million hectares, representing nearly half of global tobacco production (WNBS. PRC, 2022). However, improvements in tobacco yield and quality in China have been significantly limited in recent years due to potassium (K) deficiency in tobacco-planted soil and a comparatively high demand for K in tobacco plants (Hu et al., 2019, 2021; Lu et al., 2022). As a result, K_2_SO_4_ fertilizer, the main supplement for enhancing the soil K content and tobacco quality, is being overused by farmers in China, and which has caused great environmental harm (Arienzo et al., 2009; Zhang and Kong, 2014), affecting the soil mineral balance (Farrokh et al., 2012) and bacterial community (Song et al., 2019). Therefore, there is an urgent need to determine the optimal application amount of K_2_SO_4_ fertilizer to improve tobacco yield and quality, prevent soil health deterioration, and then achieve the sustainable and healthy development of the tobacco industry in China.

Due to its advantages of higher K content, good solubility, and low cost, K_2_SO_4_ fertilizer has become a vital component of tobacco planting recommended for farmers in China (Li et al., 2019). In general, a leaf K content above 2% is considered crucial for enhancing the processing properties of tobacco leaves and improving the burnability of cigarettes (Karbachsch, 1978). A higher K content can improve the photosynthetic performance of tobacco leaves, thus promoting dry matter accumulation and translocation (Andrés et al., 2014; Abbasi et al., 2016). K is also involved in osmoregulation, enzyme activation, and carbohydrate and protein formation (Fageria et al., 2001; Hu et al., 2019). However, there is no linear relationship between the amount of K_2_SO_4_ fertilizer applied and its effects on the K content of soil or tobacco leaves. By contrast, an excessive K content in soil resulting from K_2_SO_4_ fertilizer application often has an antagonistic effect on Ca^2+^, NH_4_^+^, and Mg^2+^ absorption (Nieder et al., 2011; Lu et al., 2022). Meanwhile, excessive soil K content has also been confirmed to induce plant salt stress, and which lead to the destruction of plant photosynthesis and subsequent yield loss (Zhu et al., 2025). Delayed tobacco maturation and limited nicotine production have also been demonstrated under excessive K_2_SO_4_ fertilizer application (Vann et al., 2012).

Sulfur (S) is also an essential nutrient for tobacco growth, and it is mainly absorbed from the soil. It has been suggested that crop nutrient uptake generally increases with increasing soil availability. The S content in tobacco leaves is recommended to be below 0.67%, which is crucial for the combustion of cigarettes (Hu et al., 2020). Soil acidification has been confirmed after long-term K_2_SO_4_ fertilizer application when an excessive amount of SO_4_^2−^ is applied to tobacco-planted soil (Xu and Ji, 2001). The Ca^2+^ and Mg^2+^ contents are also affected by soil residual SO_4_^2−^ through the formation of CaSO_4_ and MgSO_4_ compounds (Jiang et al., 2024). Moreover, the bacterial community and functional pathways of tobacco-planted soil are destroyed when the K_2_SO_4_ fertilizer application amount is above the recommended level (Wang et al., 2021). Maintaining S levels within the optimal range in tobacco-planted soil is essential for tobacco yield and quality.

The balance between different soil mineral composition is crucial for improving plant growth. It is generally believed that antagonism and synergism are common in ion absorption (Bakker et al., 2013). Besides, the ratio of ion content, such carbon-to-nitrogen ratio, is also essential for enhancing plant yield and quality (Cheng et al., 2025). A higher ammonium (NH_4_^+^) to K ratio can lead to soil acidification due to more H^+^ ions were released during the conversion of NH_4_^+^ to nitrate (NO_3_^–^) in agricultural production (Jones et al., 2019). Meanwhile, the imbalance in plant nutrient uptake also leads to more severe soil acidification (Dong et al., 2021). An optimal nitrogen to potassium ratio has also been shown to promote the maturation of tobacco leaf and enhance its quality (Schwamberger and Sims, 1991; Hu et al., 2021).

The optimal soil pH for tobacco growth is between 5.5 and 6.5 (Dai et al., 2021; Zhang et al., 2016), and the deficiency of soil phosphorus (P) and K has been demonstrated when soil is acidified (Martins et al., 2014). Tang et al. (2020) further confirmed that total sugar, reducing sugar, and K content in tobacco were negatively correlated with soil pH. Wan et al. (2020) showed that bacterial community functions, including S cycling-related enzymes and proteins, were all downregulated in acidic soils. Moreover, pH is widely considered a dominant predictor of soil microbial activity, which not only affects tobacco growth but also influences disease occurrence (She et al., 2017; Wang et al., 2019). Improving pH in tobacco-planted soil has become an important prerequisite for optimizing soil and tobacco mineral composition.

K_2_SO_4_ fertilizer application is a common choice for farmers to address K deficiency in tobacco-planted soil. Although many advantages of applying K_2_SO_4_ fertilizer on soil structure, mineral composition, and tobacco growth have been verified (Vann et al., 2013; Zhang and Kong, 2014), the optimal application amount and matched-use technologies are still unclear in Shandong, especially in long-term fertilization experiments. Optimizing the K_2_SO_4_ fertilizer application amount is necessary to achieve healthy soil and high-quality tobacco leaves, and the application amount may vary among tobacco-growing regions. Farmers need an optimal recommended amount when applying K_2_SO_4_ fertilizer in the field.

We hypothesized that changing the K_2_SO_4_ fertilizer application amount would affect soil physicochemical properties and then impact tobacco tissue K content and dry matter accumulation. Therefore, the objectives of this study were (a) to quantify the effects of various K_2_SO_4_ fertilizer application amounts on soil pH, mineral composition, and soil-related enzyme activity and to compare the above values with the initial soil physicochemical properties at 10 years ago; (b) to investigate the relationship between tobacco total matter accumulation, tissue K content and soil physicochemical characteristics; and (c) to determine the optimal K_2_SO_4_ fertilizer application amount for both tobacco quality enhancement and soil health.

## 2 Materials and methods

### 2.1 Experimental site

**Figure 1:**
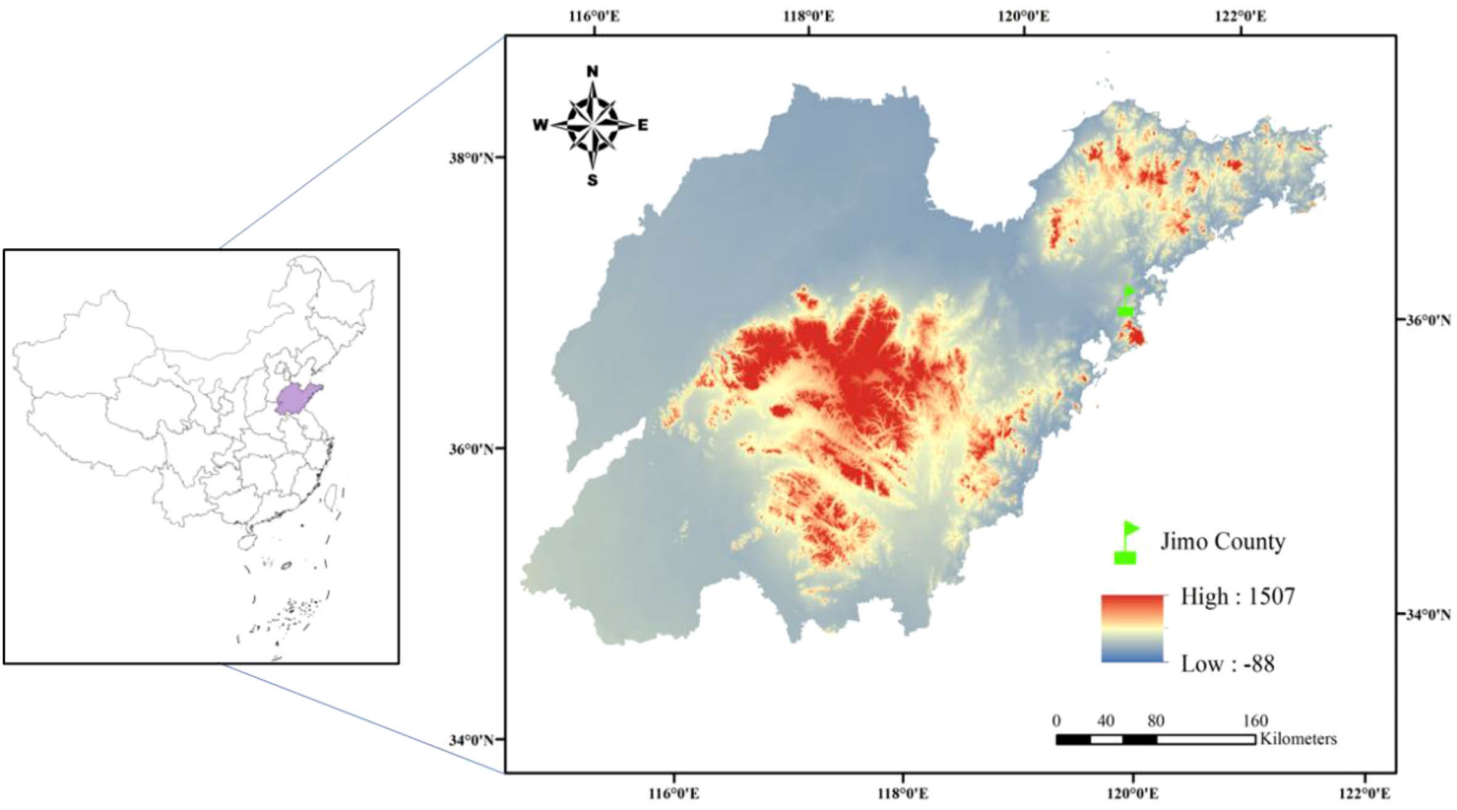
Shandong Province and the long-term fertilization experiment site located in Qingdao, China.

**Figure 2:**
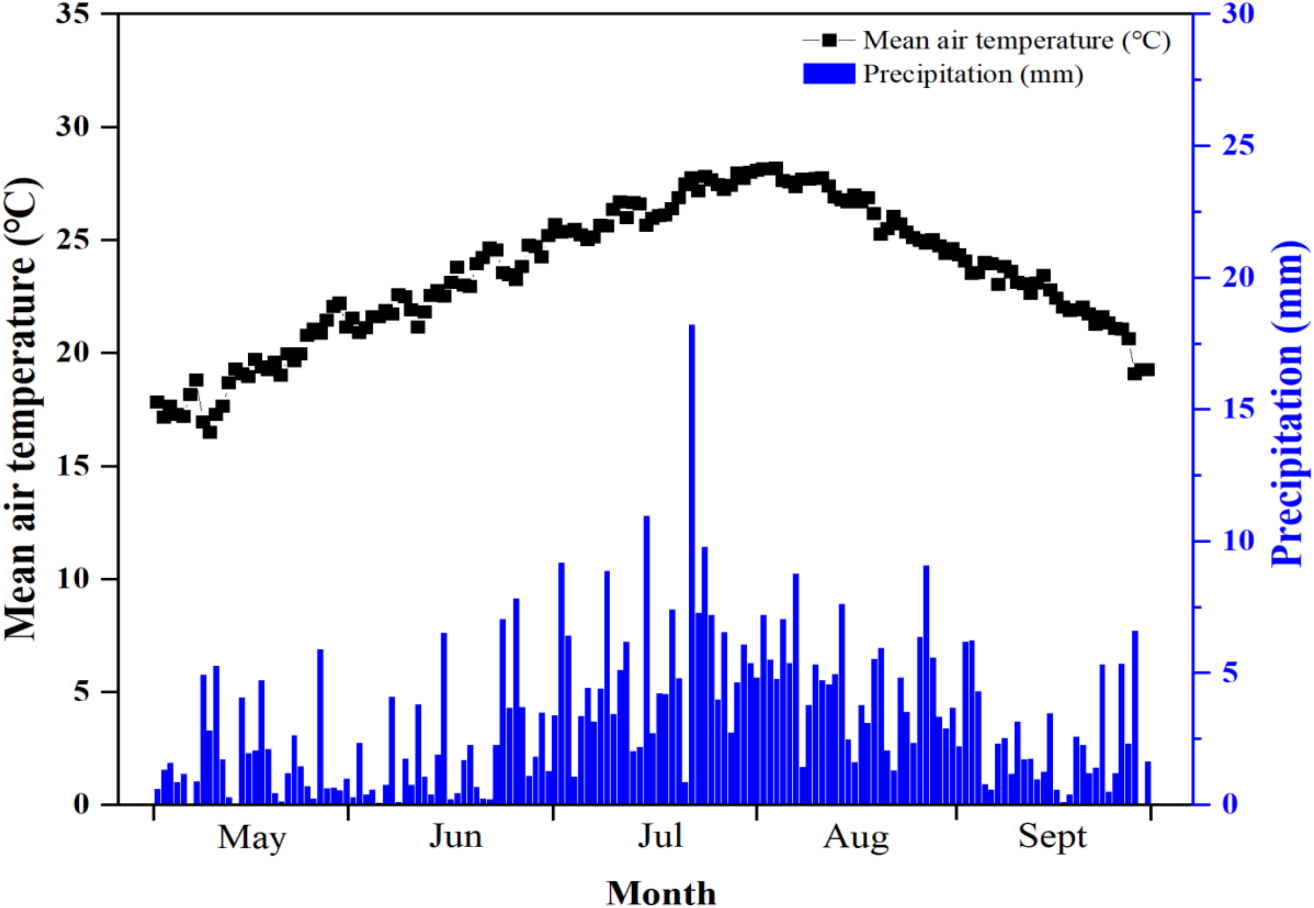
Daily mean air temperature and precipitation during tobacco-growing seasons in Qingdao, China, from 2010 to 2021.

The long-term (2010–2021) fertilization experiment was carried out in Jimo County (36°27’ N, 120°35’ E), Qingdao City, Shandong Province, China, within the warm temperate monsoon continental climate region (**Figure 1**). The soil at the experiment site is Alfisols according to the FAO Soil Taxonomic System. The initial soil organic matter, total nitrogen (N), total K, available N (AN), available K (AK), and pH in the top 0–20 cm soil layer in 2010 were 11.7 g kg^−1^, 0.87 g kg^−1^, 15.8 g kg^−1^, 52.7 mg kg^−1^, 105.3 mg kg^−1^, and 5.6, respectively. In addition, the ammonium nitrogen (NH_4_^+^–N) and nitrate nitrogen (NO_3_^−^–N) contents were 5.5 and 9.3 mg kg^−1^, respectively. The weather conditions during the tobacco-growing season (May–September) in Jimo from 2010 to 2021 are shown in **Figure 2**. The daily average air temperature was approximately 13.7°C, and the mean annual rainfall was 643.7 mm. The weather data were retrieved from the WheatA System (http://www.wheata.cn/).

### 2.2 Experimental design and management

**Figure 3:**
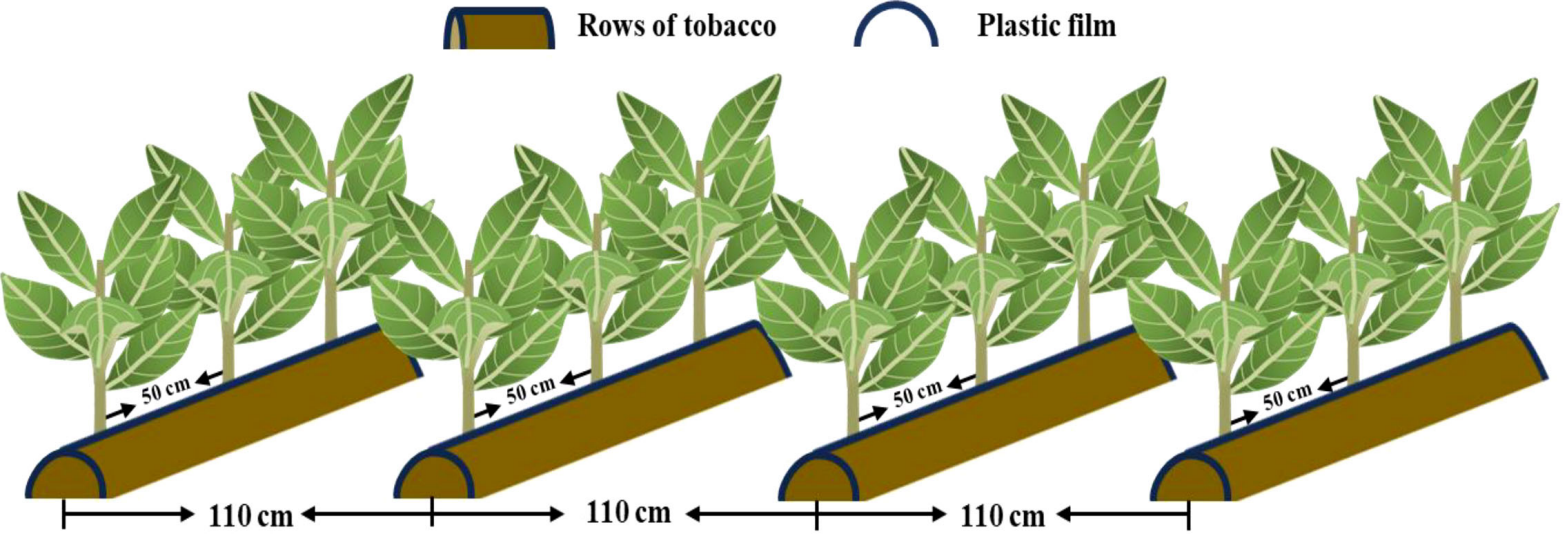
The cultivation pattern of tobacco plants per plot in the long-term fertilization experiment in Qingdao, China.

The long-term fertilization experiment, which started in 2010, was arranged in a completely randomized block design with 3 replicates of each treatment. Each plot contained 4 rows of tobacco, with a plant spacing of 50 cm and a row spacing of 110 cm. The ridge width was fixed at 60 cm. The total plot area was 22.0 m^2^ (5 m long × 4.4 m wide), including 40 tobacco plants. Concrete walls were adopted to separate each plot from the others to prevent fertilizer interactions and the irrigation was applied with drip irrigation in each plot (**Figure 3**). The experiment consisted of 4 fertilization treatments based on results of the pre-test and local typical farmer practices: CK (no fertilizer); K_82.5_+S_66_ (550 kg ha^−1^ potassium sulfate compound fertilizer with 15% N, 15% P_2_O_5_, 15% K_2_O, and 12% S); K_165_+S_95.7_ (165 kg ha^−1^ K_2_SO_4_ with 50% K_2_O and 18% S added to K_82.5_+S_66_); and K_247.5_+S_125.4_ (330 _kg_ ha^−1^ K_2_SO_4_ added to K_82.5_+S_66_). The specific annual fertilizer application amount applied in each plot is shown in **Table 1**. The tobacco cultivation pattern used in this study was continuous cropping.

**Table 1:** Specific annual application amount of nutrient (kg ha^−1^) in the long-term fertilization experiment (2010–2021).

Treatments	N:P:K	N	P_2_O_5_	K_2_O	S
CK	—	0.0	0.0	0.0	0
K_82.5_+S_66_	1:1:1	82.5	82.5	82.5	66.0
K_165_+S_95.7_	1:1:2	82.5	82.5	165.0	95.7
K_247.5_+S_125.4_	1:1:3	82.5	82.5	247.5	125.4

K and S in the first column represent K_2_O and S, respectively, and the subscript number represents the application amount (kg ha^−1^).

Tobacco cultivar ‘NC89’ was transplanted in mid-May and harvested in early September. Tobacco was seeded by hand in greenhouses and then transplanted to the field in hills. The N, P, and K fertilizers were applied before soil plowing in each tobacco season with a compound fertilizer with 15% N, 15% P_2_O_5_, 15% K_2_O, and all K_2_SO_4_ fertilizer (K_2_O, 50%). All basal fertilizers were mixed with 0–20 cm soil, and tobacco seedlings were transplanted after ridging. All crop biomass was removed from each plot following the tobacco harvest. In addition, manual topping (removing the growth terminals on the main stems manually) and inhibition of tobacco axillary buds were conducted throughout the tobacco-growing season. Other management practices, such as weed control and insect scouting, were conducted in a timely manner in each plot according to local standard tobacco agronomic practices.

### 2.3 Sample collection and measurements

The soil in the plow layer (0–20 cm) was sampled according to the “S” type following tobacco harvest on September 10, 2021, which was 10 years after the long-term fertilization experiment was initiated. Five soil samples were collected from each plot and mixed together to obtain one sample for replication. Visible roots, organic residues, and stone fragments in soil samples were removed manually before measurement.

Each sample was gently crushed and divided into two portions. The fresh soil sample was used to determine the NH_4_^+^–N and NO_3_^−^–N contents using a continuous flow injection analyzer (AMS Alliance, FUTURA, France) after extraction with 2 M KCl. The other soil sample was passed through a 2-mm sieve to determine the soil AN and AK contents. The AN content was quantified using the alkaline diffusion method, and soil AK was extracted with 1.0 M NH_4_OAc according to the method described by Lu (2000) and measured with a flame photometer (SHjingmi Inc., 6400A, Shanghai, China). The soil pH was determined using a pH meter with a soil-to-water ratio of 1:2.5 (Meter3100C).

Soil urease, acid phosphatase, and sucrase activities were measured using indophenol blue colorimetry (Yang et al., 2008), the disodium p-nitrophenyl phosphate colorimetry method (Jiang et al., 2021), and the 3,5-dinitrosalicylic acid colorimetric method (Han et al., 2012), respectively. Soil arylsulfatase activity was calculated by determining the amount of p-nitrophenol (PNP).

Three representative tobacco plants from the central rows of each plot were manually harvested throughout the tobacco-growing season in 2021 to quantify dry matter accumulation. Sampled tobacco plants were cut at the soil surface and divided into roots, stems, and leaves after washing. Samples for each organ were dried at 105°C for 2 h and then dried to a constant weight at 85°C. The tobacco dry matter was weighed with an electronic balance, and these root, stem, and leaf samples were ground to a paste and sieved with a 0.5-mm mesh. The tobacco plant K content was digested by H_2_SO_4_–H_2_O_2_ digestion and analyzed by flame spectroscopy (SHjingmi Inc., 6400A, Shanghai, China).

### 2.4 Statistical analysis

We used a one-way analysis of variance (ANOVA) to analyze all data with SPSS 21.0 (SPSS Inc., Chicago, IL, USA). Duncan’s multiple range test was adopted to separate significant differences at the 95% probability level. The relationship between the tobacco dry matter, K content and soil mineral composition was investigated using Pearson correlation in Origin 2018 (Origin Lab Co., Northampton, MA, USA). The location of the long-term fertilization experiment site was determined using ArcMap 10.8 (ESRI Inc., Redlands, CA, USA), and all data were visualized using Origin 2018.

## 3 Results

### 3.1 Soil pH

**Figure 4:**
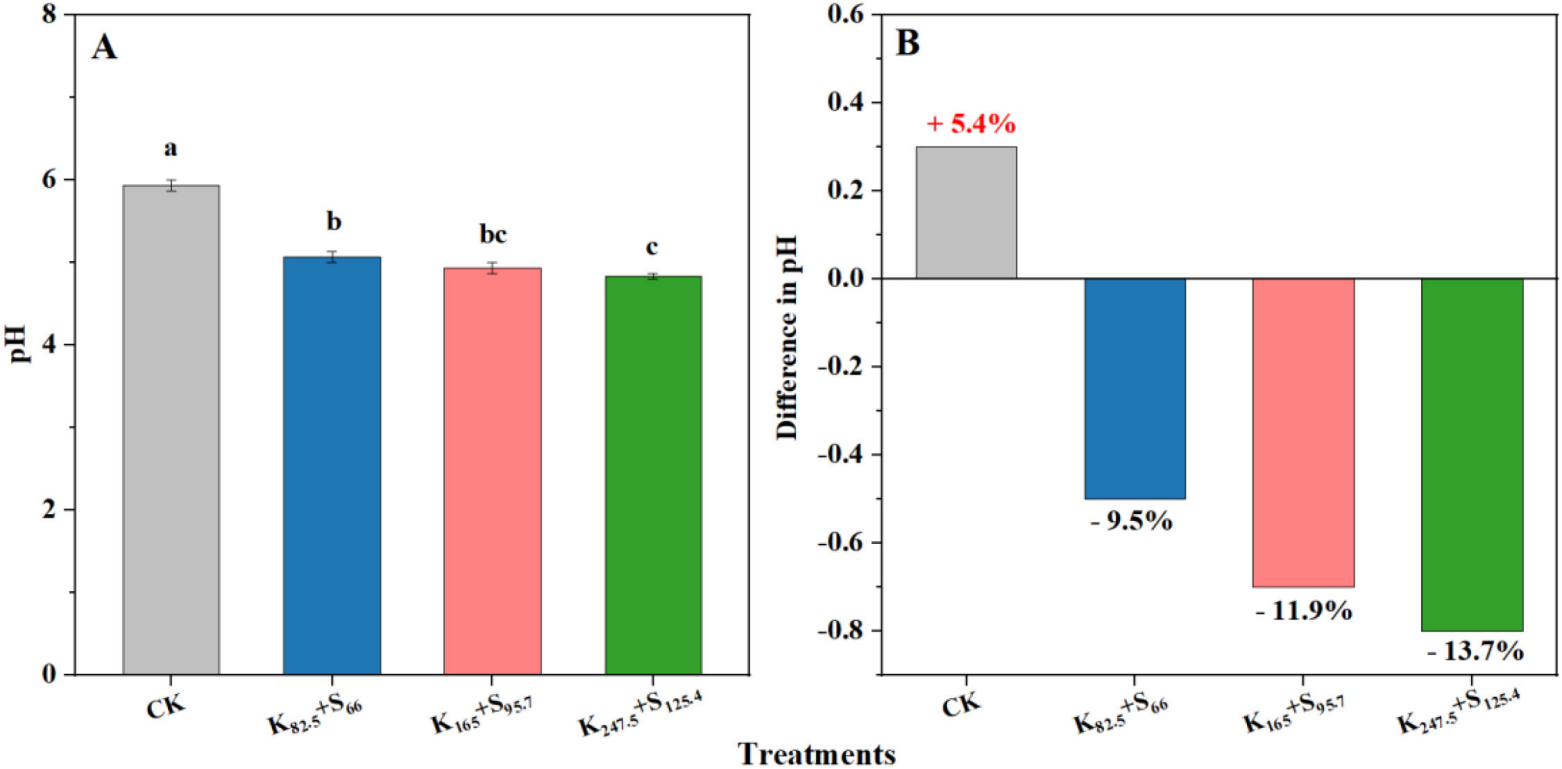
Response of soil pH to the K_2_SO_4_ fertilizer application amount in a 10-year long-term fertilization experiment. **(A)** Comparison of soil pH after long-term K_2_SO_4_ fertilizer application. Different lowercase letters above the bars indicate significant differences at *P* < 0.05. **(B)** The variation in soil pH relative to the initial soil pH at 10 years ago. The red and black percentage values represent an increase and decrease in pH, respectively. CK indicates no fertilizer. K and S indicate K_2_O and S, respectively, and the following lowercase number represents the application amount (kg ha^−1^).

Compared with CK, K_2_SO_4_ fertilizer application significantly decreased soil pH. Among the four treatments, the soil pH under CK was 5.9, which was 15.7%, 20.4%, and 22.9% higher than that under K_82.5_+S_66_, K_165_+S_95.7_, and K_247.5_+S_125.4_, respectively. Specifically, soil pH showed a decreasing trend with increasing K_2_SO_4_ fertilizer application amount. However, no significant difference was observed in soil pH among K_82.5_+S_66_ and K_165_+S_95.7_. The effects of K_165_+S_95.7_ and K_247.5_+S_125.4_ on soil pH were also similar (**Figure 4A**). Furthermore, compared with initial soil pH when the fertilization experiment was designed, the soil pH under K_82.5_+S_66_, K_165_+S_95.7_, and K_247.5_+S_125.4_ decreased by 9.5%, 11.9%, and 13.7%, respectively, over the past 10 years. By contrast, the soil pH under CK increased by 5.4% compared to the initial pH (**Figure 4B**). The rapid decrease in soil pH caused by long-term excessive K_2_SO_4_ fertilizer application aggravated the acidification of tobacco-planted soil.

### 3.2 Soil potassium content

**Figure 5:**
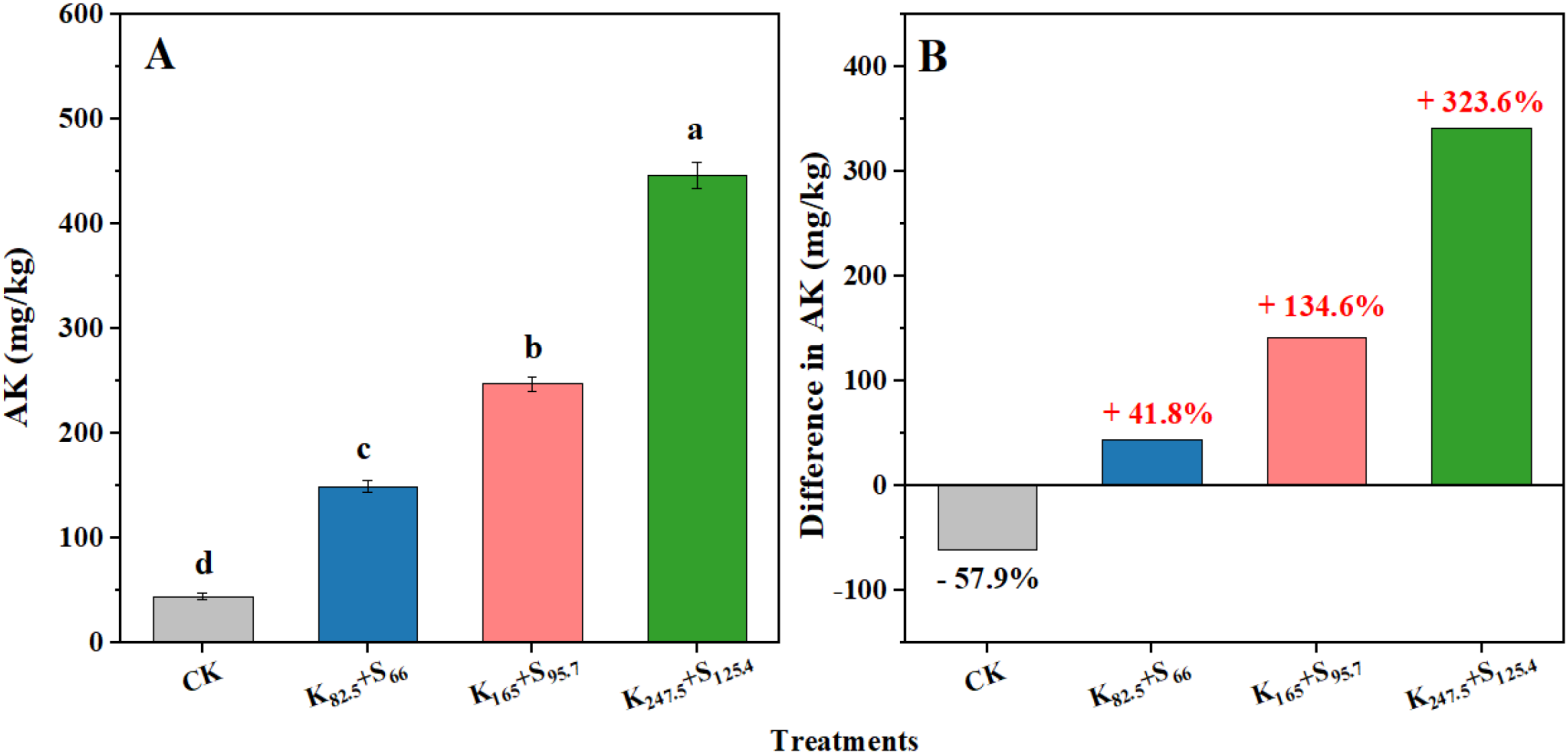
Soil available potassium (AK) as affected by long-term K_2_SO_4_ fertilizer application in a 10-year fertilization experiment. **(A)** Content of soil AK. Different lowercase letters indicate significant differences at the 0.05 probability level. **(B)** The variation in soil AK compared to the initial value at 10 years ago. The red and black percentage values adjacent to the bars shown an increase and decrease in soil AK, respectively. CK means no fertilizer. K and S indicate K_2_O and S, respectively, and the following number represents the application amount (kg ha^−1^).

As shown in **Figure 5A**, compared with CK, the soil AK content significantly increased by 105.0, 202.7, and 401.8 mg kg^−1^ under K_82.5_+S_66_, K_165_+S_95.7_, and K_247.5_+S_125.4_, respectively. The significant difference in soil AK content was observed between every two treatments among the four K_2_SO_4_ fertilizer treatments. Moreover, the soil AK content increased with the amount of K_2_SO_4_ fertilizer applied, resulting in the highest soil AK content under K_247.5_+S_125.4_. Besides, the soil AK content under K_82.5_+S_66_, K_165_+S_95.7_, and K_247.5_+S_125.4_ obviously increased by 44.0 (41.8%), 141.7 (134.6%), and 340.8 (323.6%) mg kg^−1^, respectively, compared to the initial soil AK content in 2010. However, the soil AK content under CK decreased by 61.0 (57.9%) over the past 10 years due to the lack of effective K supply (**Figure 5B**). The excessive K ions was introduced to tobacco-planted soil from K_2_SO_4_ fertilizer application during the past 10 years may lead to the initial mineral compositions in soil undergo diverse responses and changes.

### 3.3 Soil nitrogen content

**Figure 6:**
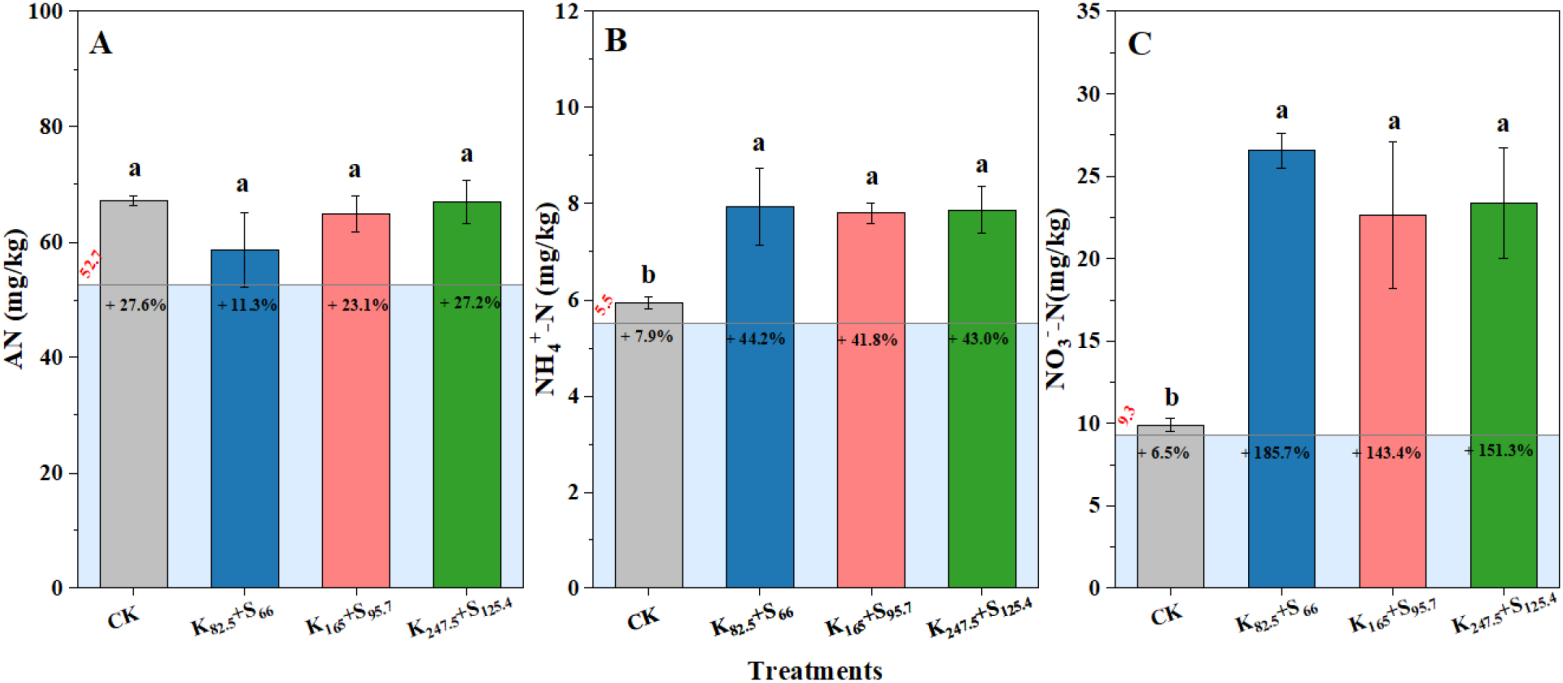
Soil nitrogen content after the 10-year long-term K_2_SO_4_ fertilizer application. **(A)** Available nitrogen (AN); **(B)** Ammonium nitrogen (NH4^+^–N); **(C)** Nitrate nitrogen (NO_3_^−^–N). The red values represent the initial soil nitrogen content at 10 years ago and the black percentage values within the bars shown the soil nitrogen variation compared to the above red values. Different lowercase letters above the bars indicate significant differences among four fertilizer treatments at the 0.05 probability level. CK means no fertilizer. K and S indicate K_2_O and S, respectively, and the following number represents the application amount (kg ha^−1^).

The effects of different K_2_SO_4_ fertilizer application amount on soil AN content were not significant. However, compared with the initial soil AN performance (52.7 mg kg^−1^), the soil AN content under CK, K_82.5_+S_66_, K_165_+S_95.7_, and K_247.5_+S_125.4_ increased by 27.6%, 11.3%, 23.1%, and 27.2%, respectively, over the past 10 years (**Figure 6A**). The response of NH4^+^–N and NO_3_^−^–N contents to K_2_SO_4_ fertilizer application was not the same as that of soil AN. Compared with CK, the soil NH4^+^–N content increased significantly by 34.4%, 32.2%, and 33.3% under K_82.5_+S_66_, K_165_+S_95.7_, and K_247.5_+S_125.4_, respectively. Meanwhile, the soil NH4^+^–N under the four K_2_SO_4_ fertilizer treatments increased by 7.9%, 44.2%, 41.8%, and 43.0%, respectively, compared to the initial NH4^+^–N content (**Figure 6B**). In addition, the soil NO_3_^−^–N content showed similar changes with NH4^+^–N, with increases of 168.4%, 128.6%, and 136.0%, respectively, compared with CK. And, the soil NO_3_^−^–N content under the above four K_2_SO_4_ fertilizer treatments increased by 6.5%, 185.7%, 143.4%, and 151.3%, respectively, compared to the initial NO_3_^−^–N content (**Figure 6C**). However, there were no significant differences in soil NH4^+^–N and NO_3_^–^N contents among K_82.5_+S_66_, K_165_+S_95.7_, and K_247.5_+S_125.4_ (**Figure 6**).

### 3.4 Soil nitrogen-to-potassium ratio

**Figure 7:**
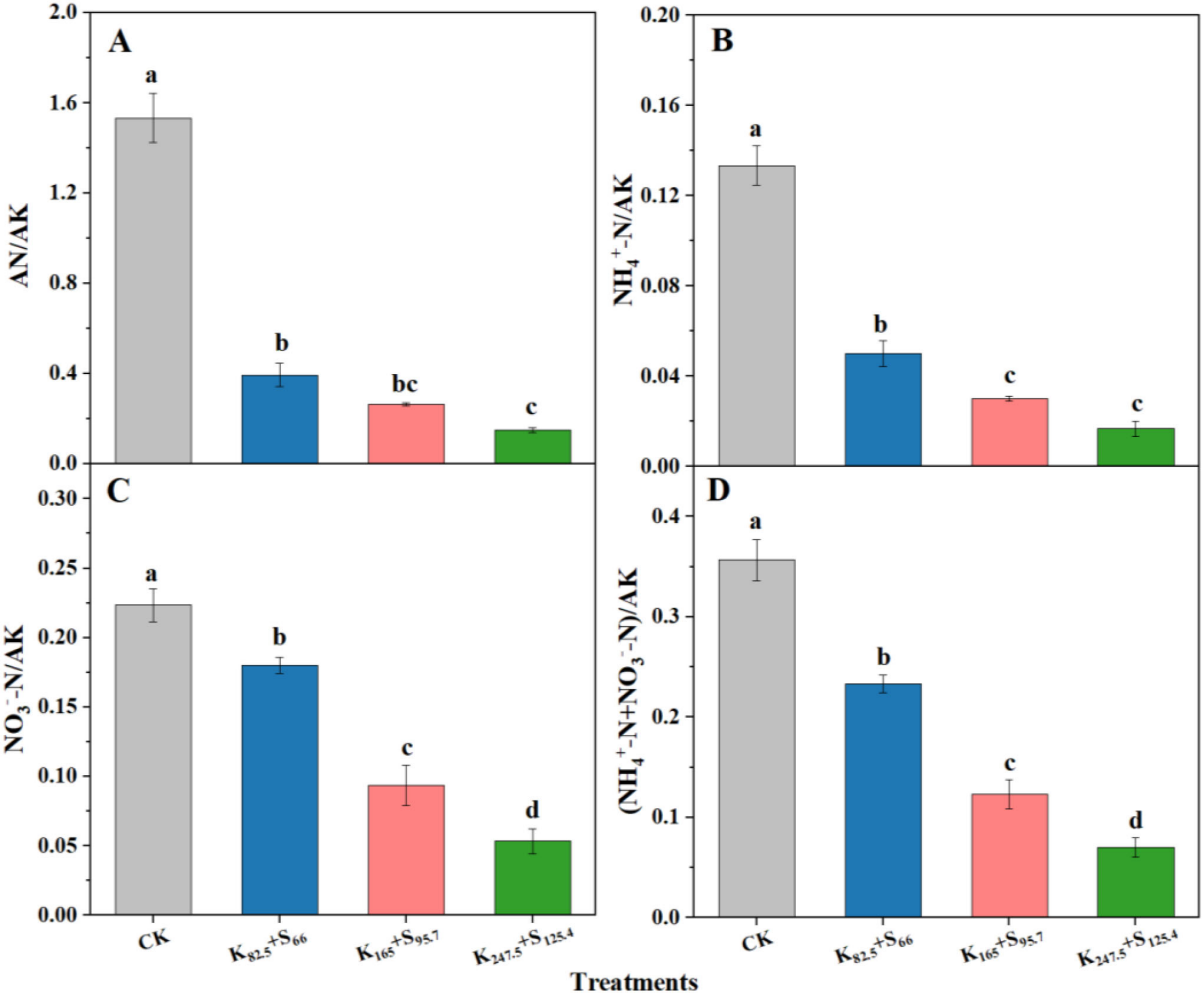
The performance of nitrogen-to-potassium ratio in relation to the long-term K_2_SO_4_ fertilizer application. **(A)** Ratio of available nitrogen (AN) to available potassium (AK). **(B)** Ratio of ammonium nitrogen (NH_4_^+^–N) to AK. **(C)** Ratio of nitrate nitrogen (NO_3_^–^–N) to AK. **(D)** Ratio of NH_4_^+^–N and NO_3_^–^–N to AK. Different lowercase letters above the bars indicate significant differences among the four fertilizer treatments at P < 0.05. CK means no fertilizer. K and S indicate K_2_O and S, respectively, and the following number represents the application amount (kg ha^–1^).

Optimizing the nitrogen-to-potassium ratio is an effective approach to improve tobacco yield and quality. As shown in **Figure 7**, the performance of AN/AK, NH4^+^–N/AK, NO_3_^–^_—_N/AK, and (NH4^+^–N+NO_3_^–^_—_N)/AK showed a similar decreasing trend with increasing K_2_SO_4_ fertilizer application amount. The highest soil nitrogen-to-potassium ratios were observed under the CK treatment. However, the difference in AN/AK between K_82.5_+S_66_ and K_165_+S_95.7_ was not significant and no marked effects of K_165_+S_95.7_ and K_247.5_+S_125.4_ on AN/AK were discovered (**Figure 7A**). Besides, there was also no significant difference in NH4^+^–N/AK between K_165_+S_95.7_ and K_247.5_+S_125.4_. The excessive K ions was introduced to soil due to K_2_SO_4_ fertilizer application reduced the nitrogen-to-potassium ratio, which potentially regulating tobacco growth and leaf quality.

### 3.5 Soil enzyme activity

**Figure 8:**
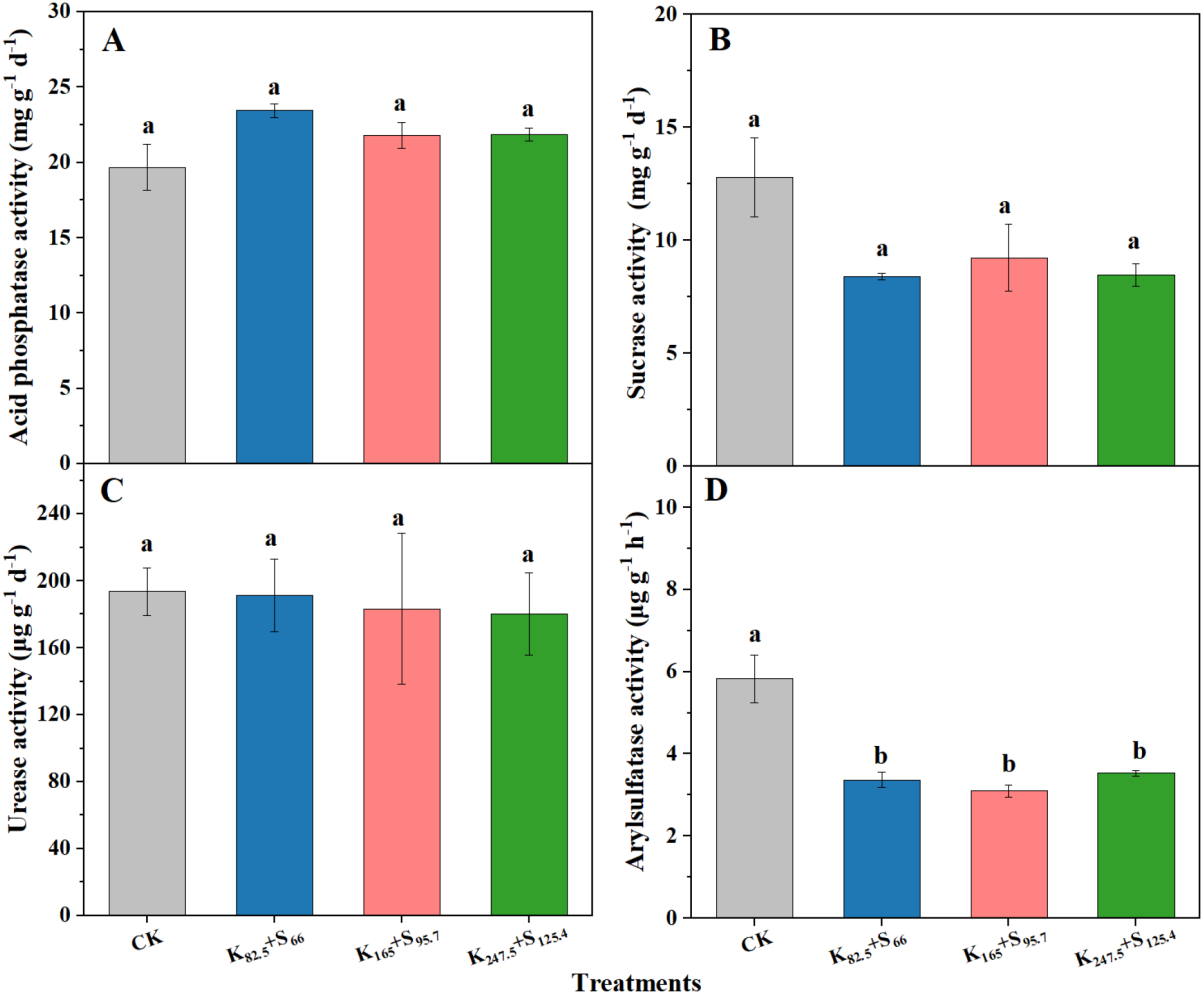
Enzyme activity of tobacco-planted soil after long-term K_2_SO_4_ fertilizer application. **(A)** Acid phosphatase activity. **(B)** Sucrase activity. **(C)** Soil urease activity. **(D)** Soil arylsulfatase activity. The same lowercase letters indicate a lack of significant difference at *P* < 0.05. CK means no fertilizer. K and S represent K_2_O and S, respectively, and the following number is the application amount (kg ha^−1^).

Soil enzyme activity, such as soil nutrient supply and soil microbial activity, reflects the health level of tobacco-planted soil. As shown in **Figure 8D**, long-term K_2_SO_4_ fertilizer application significantly affected the arylsulfatase activity, resulting in significant decreases of 42.0%, 46.6%, and 39.1% under K_82.5_+S_66_, K_165_+S_95.7_, and K_247.5_+S_125.4_, respectively, compared with CK. However, no significant differences were observed in arylsulfatase activity among the above-mentioned three K_2_SO_4_ fertilizer treatments. By contrast, the differences in acid phosphatase, sucrase, and urease activities among all four treatments including CK were not significant after long-term K_2_SO_4_ fertilizer application (**Figures 8A–C**).

### 3.6 Potassium content in tobacco tissues

The K content in tobacco roots, stems, and leaves varied similarly (**Table 2**). For example, the K content of tobacco roots increased significantly by 10.4% and 43.8% under K_165_+S_95.7_ and K_247.5_+S_125.4_, respectively, compared with CK treatment, but the differences between CK and K_82.5_+S_66_ as well as among K_165_+S_95.7_ and K_247.5_+S_125.4_ were not significant. Meanwhile, compared with CK, the K content of tobacco leaves also increased significantly by 11.9% and 17.9% under K_165_+S_95.7_ and K_247.5_+S_125.4_, respectively. The K content in tobacco roots, stems, and leaves increased with the K_2_SO_4_ fertilizer application amount, and the K content was highest in tobacco leaves. Among the four treatments, the average K content was 162.3% and 45.2% higher in tobacco leaves than that in tobacco roots and stems, respectively. Overall, the K_165_+S_95.7_ application amount was more reasonable compared with the other treatments in terms of utilization and economy (**Table 2**).

**Table 2:** Potassium content of tobacco roots, stems, and leaves after the long-term K_2_SO_4_ fertilizer application in 2021.

Treatments	Potassium content (g kg^−1^)		
	Root	Stem	Leaf
CK	4.8 ± 0.12 c	7.7 ± 1.15 b	13.4 ± 0.32 b
K_82.5_+S_66_	4.8 ± 0.03 c	9.7 ± 0.75 ab	13.0 ± 1.18 b
K_165_+S_95.7_	5.3 ± 0.20 ab	11.0 ± 0.15 a	15.0 ± 0.58 a
K_247.5_+S_125.4_	6.9 ± 0.12 a	11.0 ± 1.00 a	15.8 ± 0.12 a

The same lowercase letters within same column indicate no significant differences between treatments at *P* < 0.05. CK means no fertilizer. The K and S indicate K_2_O and S, respectively, and the next number represent the application amount (kg ha^−1^).

### 3.7 Accumulation and distribution of dry matter

Compared with CK, the total dry matter of tobacco plants showed a significant difference in the response to the three K_2_SO_4_ fertilizer application amounts over course of 10 years, with increases of 64.6%, 69.3%, and 48.0% under K_82.5_+S_66_, K_165_+S_95.7_, and K_247.5_+S_125.4_, respectively. Furthermore, the increasing trend in tobacco total dry matter gradually weakened when long-term excessive amounts of K_2_SO_4_ fertilizer were applied under K_247.5_+S_125.4_. Similarly, the dry matter of tobacco stems under K_82.5_+S_66_ and K_165_+S_95.7_ demonstrated substantial enhancements compared with that under K_247.5_+S_125.4_, showing significant increases of 77.7% and 96.1%, respectively, compared with CK. The dry matter of tobacco leaves that as the harvested organ increased with the amount of K_2_SO_4_ fertilizer applied, but the differences among the four treatments were not significant. Besides, K_2_SO_4_ fertilizer application amount also promoted dry matter accumulation in tobacco roots under K_82.5_+S_66_ and K_165_+S_95.7_ compared with the other two treatments (**Table 3**). The tobacco dry matter accumulation under CK treatment was seriously declined due to insufficient nutrient supply.

**Table 3:** Dry matter accumulation and distribution in tobacco plants in 2021 as affected by long-term K_2_SO_4_ fertilizer application.

Treatments	Dry matter (g plant^−1^)			Total biomass
	Root	Stem	Leaf	
CK	27.1 ± 5.3 c	60.9 ± 12.9 b	32.9 ± 3.2 a	122.9 ± 20.3 b
K_82.5_+S_66_	54.8 ± 1.3 a	108.2 ± 8.9 a	37.4 ± 2.5 a	202.3 ± 8.8 a
K_165_+S_95.7_	47.8 ± 3.4 ab	119.4 ± 4.9 a	38.9 ± 4.4 a	208.1 ± 11.0 a
K_247.5_+S_125.4_	42.9 ± 2.0 b	97.2 ± 4.9 a	39.9 ± 3.8 a	181.9 ± 9.6 a

Different letters within same column indicate significant differences at the 0.05 level using Duncan’s multiple range test. CK means no fertilizer. K and S indicate K_2_O and S, respectively, and the following number is the application amount (kg ha^−1^).

### 3.8 Relationships between soil physicochemical properties and tobacco growth

**Figure 9:**
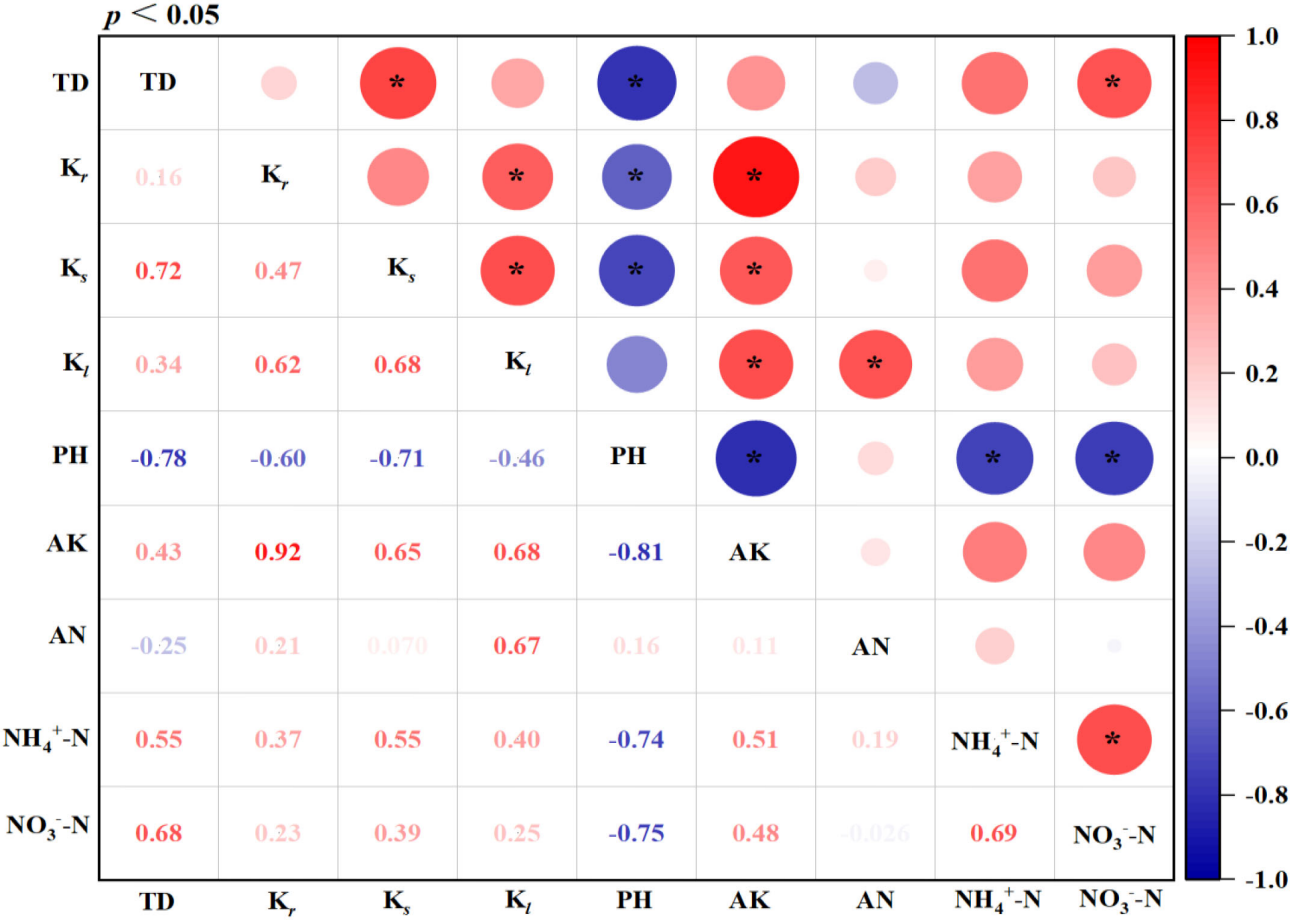
Correlation analysis of soil physicochemical properties, total dry matter (TD), and the K content of tobacco roots (K*_r_*), stems (K*_s_*), and leaves (K*_l_*) after long-term K_2_SO_4_ fertilizer application. AK and AN represent soil available potassium and available nitrogen, respectively. The asterisk (*) denotes a significance.

As shown in **Figure 9**, the K content in tobacco roots (K*_r_*), stems (K*_s_*), and leaves (K*_l_*) showed a significant positive correlation with the soil AK, with correlation coefficients higher than 0.6. However, soil pH significantly reduced the K*_r_* and K*_s_*, resulting in a negative correlation. In addition, the pH of tobacco-planted soil showed a significant negative correlation with tobacco total dry matter (TD), soil AK, NH4^+^–N, and NO_3_^−^–N contents. The K contents in tobacco roots (K*_r_*), stems (K*_s_*), and leaves (K*_l_*) were also significantly and positively correlated with each other. Moreover, the NH4^+^–N content showed a significant positive correlation with the NO_3_^−^–N content. The tobacco total dry matter (TD) was also significantly affected by soil pH, K*_s_* and the NO_3_^−^–N content. Besides, the K content in tobacco leaves (K*_l_*) was also significantly enhanced with increasing the soil AN content. In all, the change in soil pH caused by long-term excessive K_2_SO_4_ fertilizer application not only deteriorated soil physicochemical properties but also significantly reduced dry matter and K uptake in tobacco plants.

## 4 Discussion

The physicochemical properties and microbial communities in soil which serve as the foundation for crop growth are affected by crop species, fertilizer application amount, and the local ecological environment (Eslaminejad et al., 2020; Shao et al., 2020; Zhang et al., 2023a). Shandong is located in northern China, and has become a representative tobacco-growing region in the country (**Figure 1**). The soil types and climate characteristics shows regional features. Besides, fertilizer application is widely recommended for tobacco production due to its many advantages. As a result, the long-term application of K_2_SO_4_ fertilizer in tobacco cultivation may lead to the unique soil physicochemical properties and diverse performance of dry matter accumulation in Shandong. Previous studies have confirmed the decline in tobacco-planted soil quality, especially soil acidification, is becoming increasingly urgent due to K_2_SO_4_ fertilizer application (Dai et al., 2021; Lu et al., 2022). Soil pH is recognized as a pivotal predictor of soil health, which not only affects soil microbial activity and composition but also regulates soil enzyme activity (Wan et al., 2020). In this study, we further confirmed that K_2_SO_4_ fertilizer application significantly reduced the soil pH, and found which was decreased by ~10% with the long-term K_2_SO_4_ fertilizer addition over the past 10 years (**Figure 4**). The possible reasons for this performance were mainly attributed to K_2_SO_4_ fertilizer application increasing the introduction of not only K^+^ but also SO_4_²^−^ into the soil (Li et al., 2019). However, tobacco absorbs much more K^+^ than SO_4_²^−^, resulting in the accumulation of residual SO_4_²^−^ in soil. Therefore, in order to maintain charge balance, the tobacco roots secrete H^+^ to replace K^+^ (Wallace, 1994). Meanwhile, the residual SO_4_²^−^ in soil forms precipitate with Ca^2+^ and Mg^2+^ and is leached from the soil, leading to a further reduction in the content of base ions. Besides, the significantly inhibited soil acid buffer capacity due to excessive K_2_SO_4_ fertilizer use may serve as another key reason (Dong et al., 2022). In the long term, excessive application of K_2_SO_4_ fertilizer to achieve favorable tobacco yield and quality is unsustainable from the perspective of soil health.

Most K uptake by plants comes from the soil, which is partially determined by the fertilizer application amount. Therefore, farmers often hold the misconception that more fertilizer application amount leads to higher yield and benefits in production. As a result, K_2_SO_4_ fertilizer has been overused for decades in tobacco cultivation (Lu et al., 2022; Zhang et al., 2025). However, excessive K nutrient beyond what plants can uptake will lead to some disadvantages, such as salt stress and soil nutrient imbalance (Vann et al., 2012; Zhu et al., 2025). Previous research on the regulation of soil physicochemical properties and microbial communities focuses on KCl fertilizer (Grant and Bailey, 1994). In this study, significant increases in soil AK and tobacco tissue K contents were confirmed after the long-term K_2_SO_4_ fertilizer application, and the initial soil AK when the experiment was designed increased by more than 100% under K_165_+S_95.7_ and K_247.5_+S_125.4_ (**Figure 5**). However, although soil AK was positively correlated with tobacco tissue K content, there was no linear relationship between the amount of K_2_SO_4_ fertilizer applied and tobacco leaf K content (**Figure 9**, **Table 2**). The K content in tobacco leaves no longer increased when the application amount of K_2_SO_4_ fertilizer exceeds K_165_+S_95.7_ (**Table 2**). By contrast, a large amount of residual K^+^ and SO_4_²^−^ in soil due to excessive use of K_2_SO_4_ fertilizer becomes the main cause of soil acidification (Jakobsen, 1993). However, the soil AN content in our study is not consistent with that observed in previous studies, which showed a higher AN level when excessive K_2_SO_4_ fertilizer was applied (Lu et al., 2022; **Figure 6**). A possible explanation is that tobacco roots are not completely damaged when excessive K_2_SO_4_ fertilizer is used, enabling tobacco roots to absorb AN from soil. Moreover, the excellent transferability of nitrogen nutrition in soil also weakened the effects of K_2_SO_4_ fertilizer application amount on soil AN content.

Soil enzyme activity is a key factor affecting soil quality and is involved in the decomposition of organic matter, the cycling of nutrients, and the regulation of plants growth (Liu et al., 2021; Zhang et al., 2023b). Soil urease and acid phosphatase are mainly involved in the metabolism of soil nitrogen and the transformation of phosphorus, respectively (Cantarella et al., 2018). Meanwhile, soil arylsulfatase performs a crucial function in the transformation of soil available sulfur (Tabatabai and Bremner, 1970). Generally, the performance of soil enzyme activity is considered to be regulated by the soil physicochemical properties. In this study, the changes of urease activity were consistent with that of soil AN (**Figures 6**, **8**). However, it is unfortunate that we did not further investigate the variations in soil phosphorus and available carbon in this study, and thus their relationship with activities of acid phosphatase and sucrase was not explored. Excessive use of K_2_SO_4_ fertilizer led more residual SO_4_²^−^ in soil, which may disrupt S cycling-related process (Jiang et al., 2024). In this study, the activity of soil arylsulfatase was significantly decreased with increasing K_2_SO_4_ fertilizer application amount, and the main reason was attributed to the drop in soil pH caused by residual H^+^ and SO_4_²^−^ (Chen et al., 2019). In addition, the differences in soil texture and microbial community caused by long-term K_2_SO_4_ fertilizer application were also key factors affecting soil arylsulfatase activity (Stott and Hagedorn, 1980).

As an essential nutrient for tobacco yield and quality, a higher K content not only favors tobacco dry matter accumulation but also improves the processing properties of tobacco leaves. Tobacco leaves are the main location of photosynthesis and metabolic activities and have the highest K content. By contrast, tobacco roots transport most of the absorbed K to the aboveground parts, resulting in the lowest K content in the entire plant (Zheng et al., 2020). Studies have also confirmed that excessive soil AK is not utilized by the plant but reduces yield and economic benefits (Daoud et al., 2020). The K_2_SO_4_ fertilizer application amount in tobacco cultivation should be within a reasonable range. In this study, we found that the K content in tobacco leaves was significantly higher than that in roots and stems, while the total K content in the tobacco plant, including roots, stems, and leaves, no longer increased significantly when K_2_SO_4_ fertilizer was applied at rates above those in K_165_+S_95.7_ (**Table 2**). The application amount in K_165_+S_95.7_ can meet the K demands of tobacco growth while avoiding soil damage. Due to the introduction of SO_4_^2−^ and other elements under excessive K_2_SO_4_ fertilizer application, soil acidification was further aggravated (**Figure 4**), which may cause tobacco root damage or ion toxicity (Xu and Ji, 2001). Tobacco plants uptake nutrients from the soil are affected by the soil’s mineral composition. Soil Ca and Mg have been demonstrated to significantly impact tobacco dry matter accumulation (Jiang et al., 2024). In addition, soil pH has significant effects on the mineral composition of tobacco-planted soil as well as soil microbial activity and diversity, which further influence tobacco plant growth (Zeng et al., 2019). Our findings are in accordance with previous studies showing excessive K_2_SO_4_ fertilizer use may lead to the damage of tobacco root absorption capacity and ion stress, thus reducing tobacco dry matter accumulation (**Table 3**). Admittedly, the deeper mechanisms of matter accumulation during the tobacco field growth period need to be further investigated in the future from the perspectives of leaf photosynthetic characteristics and matter transformation.

Over the past few decades, fertilizers have played an irreplaceable role in improving crops yield and quality, and in addressing human hunger. However, this benefit is often a single direction that humans demand from the soil. Farmers always pursue higher yield simply by applying excessive amounts of fertilizer to the soil without paying attention to the effects of residual fertilizers on soil texture, microbial activity, and soil carbon pools. In recent years, a large area of soil is facing the risk of severe degradation in China. Therefore, the conduct of long-term fertilization experiment is of great significance for optimizing fertilizer application amount and investigating the diverse responses of soil to fertilizers, and the data is valuable and reliable. In the present study, the core role of pH in regulating soil AK and tobacco tissue K was highlighted (**Figure 9**). Moreover, we also found that the total dry matter of tobacco was significantly positively correlated with the K content in stem and soil NO_3_^–^_—_N (**Figure 9**). The above findings provide representative indicators for directly assessing the effects of long-term fertilization application on soil and crops. Unfortunately, the interaction mechanism within soil physicochemical properties was not thoroughly investigated in this study, and the further research on the crosstalk between soil ions, enzyme activity, and microbial communities is urgent in our future research.

## 5 Conclusion

Applying K_2_SO_4_ fertilizer increased the soil NH4^+^–N and NO_3_^−^–N contents and improved the AK level in tobacco-planted soil, further promoting the K content in tobacco roots, stems, and leaves and enhancing total dry matter accumulation. However, excessive K_2_SO_4_ fertilizer application decreased soil pH and arylsulfatase activity and led to soil acidification. In the long term, the initial soil AK and pH exhibited converse responses to the application of K_2_SO_4_ fertilizer, and soil pH played a dominant factor in affecting total dry matter and the K content in tobacco plants and tobacco-planted soil, showing a significant negative correlation. The feasible solution in Shandong was the mixtures of 550 kg ha^−1^ compound fertilizer with 15% N, 15% P_2_O_5_, 15% K_2_O, and 12% S and 165 kg ha^−1^ K_2_SO_4_ fertilizer with 50% K_2_O and 18% S. Our results propose the optimal application amount of K_2_SO_4_ fertilizer for maximizing soil buffering capacity and improving tobacco leaf quality through a long-term experiment, providing a reference for other tobacco-growing countries where soil AK is deficient.

## Data Availability

The original contributions presented in the study are included in the article/supplementary material. Further inquiries can be directed to the corresponding authors.
